# B cell academy of the gut: an update on gut associated germinal centre B cell dynamics

**DOI:** 10.1186/s40348-024-00180-y

**Published:** 2024-08-16

**Authors:** Christopher Wichmann, Elisa Wirthgen, Carla R. Nowosad, Jan Däbritz

**Affiliations:** 1 Department of Paediatrics, Greifswald University Medical Centre, Ferdinand-Sauerbruch-Str.1, Greifswald, MV 17457 Germany; 2 The David Rockefeller Graduate Program in Bioscience, The Rockefeller University, New York, NY USA; 3 Department of Paediatrics, Rostock University Medical Centre, Rostock, MV Germany; 4 Department of Pathology, NYU Langone Grossman School of Medicine, New York University, New York, NY USA; 5 German Centre for Child and Adolescent Health (DZKJ), Site Greifswald/Rostock, Greifswald, MV Germany

**Keywords:** Mucosal immunology, B cells, Germinal centres, Microbiome, Immunoglobulin A, Inflammatory bowel disease, Celiac disease, Nutrition, Immunoglobulin A deficiency

## Abstract

**Background:**

The gut is an environment in which the immune system closely interacts with a vast number of foreign antigens, both inert such as food and alive, from the viral, bacterial, fungal and protozoal microbiota. Within this environment, germinal centres, which are microanatomical structures where B cells affinity-mature, are chronically present and active.

**Main Body:**

The functional mechanism by which gut-associated germinal centres contribute to gut homeostasis is not well understood. Additionally, the role of T cells in class switching to immunoglobulin A and the importance of B cell affinity maturation in homeostasis remains elusive. Here, we provide a brief overview of the dynamics of gut-associated germinal centres, T cell dependency in Immunoglobulin A class switching, and the current state of research regarding the role of B cell selection in germinal centres in the gut under steady-state conditions in gnotobiotic mouse models and complex microbiota, as well as in response to immunization and microbial colonization. Furthermore, we briefly link those processes to immune system maturation and relevant diseases.

**Conclusion:**

B cell response at mucosal surfaces consists of a delicate interplay of many dynamic factors, including the microbiota and continuous B cell influx. The rapid turnover within gut-associated germinal centres and potential influences of an early-life window of immune system imprinting complicate B cell dynamics in the gut.

## Introduction

The gut is an extremely complex environment. A single layer of epithelial cells separates a myriad of bacteria, viruses, protozoa, fungi, and otherwise foreign proteins from approximately 5 × 10^10^ immune cells [1]. The difficult task of the intestinal immune system is to maintain tolerance to irrelevant food antigens and support a nuanced reaction to the resident microbiome (commensals), while retaining the ability to react to intestinal infection as needed [2, 3]. B cells, as part of the adaptive immune system, can perform this task in a responsive, antigen-specific manner by producing immunoglobulin (Ig), responding to pathogens whilst tolerating and even promoting the presence of commensal bacteria [4, 5]. Among Ig isotypes, the mucosal IgA isotype is the most abundantly produced, with multiple grams generated each day in humans [2]. B cells are therefore important for the regulation of the microbial community [2, 4], and their role in diversifying the commensal microbiome has been suggested as an important evolutionary advantage in mammals [6].

B cells have a surface-bound copy of their Ig, known as the B cell receptor (BCR) [7], which is diversified in two distinct ways: recombination of variable- (V-), diversity- (D-), and heavy chain joining- (J-) segments and somatic hypermutation (SHM) [8]. Whilst V-D-J recombination happens during B cell development, SHM and subsequently affinity maturation occurs in microanatomical structures termed germinal centres (GC) [8]. Within GCs, B cells undergo iterative rounds of positive selection by CD4^+^ T follicular helper cells, subsequent division and SHM [8, 9]. Through SHM, mutations are introduced preferentially into the variable regions of Ig genes by Activation-induced Cytidine Deaminase (AID) [8]. AID deaminates cytidine to uracil, leading to mismatches. Repair of these mismatches by error-prone DNA polymerase creates mutations [8]. If a clone acquires affinity-enhancing mutations and can secure T cell help, in combination with other surrounding factors, it undergoes massive proliferation in what is called a clonal burst [7, 10]. To assume their effector functions as plasmablasts (PB), plasma cells (PC), or memory B cells, B cells can exit GCs in a process that is dependent on BCR affinity and other partially unknown signals [8, 10]. PB and PC in the lamina propria (LP) produce IgA, which is secreted into the intestinal lumen across the epithelium, where it can act on resident microbiota and regulate gut homeostasis [3, 4, 6].

### Chronic gut associated germinal centres have rapid turnover and show signs of selection

In most lymphoid organs, GCs form upon immunisation or infection, and most of our understanding of B cells and GCs has been generated under these circumstances [3]. GCs are chronically present in the mesenteric lymph nodes (mLN) and Peyer’s patches (PP) [5, 10]. While this chronic presence of gut-associated GCs (gaGC) could be explained by constant exposure to antigens from the microbiota and diet, they are also present in germ-free (GF) mice that lack a microbiome. Furthermore, they were described in the absence of antigen-binding BCRs, where B cell survival is ensured through Epstein-Barr virus latent membrane protein 2A [10–12]. Mucosal B cells prefer IgA which is produced as a dimer with a joining chain. During secretion across the mucosal epithelium a secretory component is added that comprises a cleaved portion of the polymeric Ig receptor which facilitates transport across the epithelium [3]. The isotype choice towards IgA is predominantly observable in PP GC B cells and seems to be dependent on the microbiota, since IgA^+^ GC B cells are nearly completely absent in gaGCs of GF mice [11].

Long-lasting GCs have also been observed in contexts other than gut-associated lymphoid tissue (GALT), such as infection with human immunodeficiency viruses, influenza, and severe acute respiratory syndrome coronavirus 2 [13, 14]. At later time points within these GCs, researchers could observe invasion of the GC niche by B cells with low or no detectable binding to the original GC-inducing antigen(s) [13, 14]. This might contribute to the diversification of ongoing GC responses through recruitment of low-affinity clones [14] or may be part of a reaction against other antigens utilizing the same GC structures as the initial response [13].

Similarly, within gaGCs under homeostatic conditions there is an ongoing turnover of GC B cells with GC half-lives estimated to be around two weeks using GC fate mapping approaches [11]. Utilising a multicolour fate mapping system to visualize GC selection by changes in colour composition [10], it has also been shown that gaGCs select specific B cells in a ‘clonal burst’ fashion, where one B cell clone expands massively and drives out other inferior B cell clones [10, 11]. These clonal bursts progress over time and can delay clonal turnover [11]. In a more defined setting, using reversible colonisation with a specifically designed auxotrophic bacterial strain unable to survive for prolonged periods in the mouse intestine, Hapfelmeier et al. showed GC induction lasting from two to six weeks [15]. Interestingly, they also found that there was no decrease in the IgA response targeting auxotrophic bacteria unless they subsequently colonised mice with other commensals or repeated the experiment in mice with a defined microbiome. In both of the latter cases the anti-auxotrophic IgA response decreased over time [15]. It is important to note that while the experimental setup by Nowosad et al. used stably colonised mice, Hapfelmeier et al. depended on reversibly colonising adult mice, which might influence the kinetics of the respective response [3, 11, 15].

Another factor of gaGC dynamics might be the influences of early life derived B cells. By fate mapping B cells during different time points in early life, it was shown that around half of the IgA^+^ PCs found in the small intestinal LP of mice were derived from an early post-natal development window before weaning. Likewise, within PP GCs around 16% were fate mapped, marking them as early life derived. It was furthermore shown that the response to early life rotavirus infection uses specific B cell clones, whilst the same infection in adult mice relied on different clones coming from naïve adult B cells [16]. This clearly shows, that under homeostatic conditions early life derived B cells contribute to not only the IgA^+^ PC pool, but also the PP GC B cell pool [16]. During breast feeding milk derived IgA supports microbiome homeostasis and might limit exposure of the neonatal immune system to microbiota derived stimuli. Consequently, in mice IgA^+^ PC accumulate during weaning at around 21–28 days. In humans IgA^+^ PC accumulate progressively from around 1 month after birth to 2 years of age [3]. This is reflected in a rapidly shifting microbiota in newborns, that stabilises at around the age of 3 years to resemble an adult microbiome [17]. These times might correspond to the window described above [3] and instruct B cells that are important under homeostatic conditions. The influence of an early life developmental window further complicates the selection dynamics in steady state gaGCs.

Despite this complex dynamics, it was well described that gaGCs could mount specific responses to infection, colonisation, and oral immunisation, although the role of gaGCs under homeostatic conditions was less well defined [3, 11, 18].

### Under physiological conditions most B cells in the gut are T cell dependent and GC derived

An important question is whether the generation of IgA is largely T cell-independent or T cell-dependent and is therefore generated in gaGCs. It is known that B cells can switch to IgA independently of T cells in isolated lymphoid follicles (ILF) and in the LP through a variety of signals, such as transforming growth factor β, transmembrane activator, and calcium-modulating ligand interactor among others [2, 5].

When measuring the capacity of antibodies to coat the bacterial surface, Bunker et al. found no difference in IgA coating of intestinal bacteria between T cell- or GC-deficient mice and their littermate controls [19]. Macpherson et al. showed a T cell-independent, yet antigen-specific IgA response limited to the mucosa, looking at the reactivity of intestinal IgA to commensal bacteria, outer membrane proteins, and even an introduced novel antigen [20]. However, both groups observed a reduction in IgA^+^ LP PC in the absence of gaGCs [19, 20] and intestinal and systemic IgA responses to oral immunisation was only observed in mice with functional T cells [20].

Using an oral immunisation setup depending on cholera toxin (CT), Biram et al. showed that extensive T cell help constituted an advantage for B cells in entering PP GC reactions [21]. The entry of B cells into GCs is dependent on their BCR affinity towards the NP antigen, and T cell – B cell interactions are indispensable for GC formation [21]. Lastly, in a set of experiments focusing on the role of forkhead box P3 (Foxp3) regulatory T cells (Treg), Kawamoto et al. showed that eliminating this subset of T cells in mice reduced IgA^+^ PC numbers in the small intestine and reduced microbiota diversity within the animals [22].

Thus, while the production of IgA^+^ B cells is conserved even under conditions of severe T cell deficiency, under physiological conditions (i.e., in the presence of functional T cells), most IgA^+^ PC are highly mutated, T cell-dependent, and GC-derived in order to contribute to the regulation of the bacterial community [2, 3, 5, 23].

The difference between GC-derived and GC-independent B cell development might play a role in the generation of autoantibodies in celiac disease. IgA autoantibodies against transglutaminase 2 (TG2) are a hallmark for celiac disease and patients display a massive influx of LP PC cells [24]. Contrary to responses against pathogens, SHM levels in these autoantibodies are generally low and even the usage of germline encoded sequences was observed which would indicate a GC-independent B cell development. Reversion of mutations, where observed, nonetheless led to the loss of binding in anti-GD2 antibodies [24] which is indicative of antigen-driven development occurring in GCs. Therefore, there might be differences in B cell activation leading to either entry into GCs and acquisition of mutations or GC independent activation. It is further believed that B cells also play a role as antigen presenting cells due to cross-presentation of antigen taken up by the BCR [24].

### Gut B cell specificity is detectable in oral immunisation, monocolonisation and gnotobiotic settings

Another area of active discussion is whether steady-state gaGCs select B cell clones in an antigen-specific manner. This is connected to findings detailing that B cells harbouring BCRs that bind irrelevant antigens enter gaGCs [25], that polyreactivity was frequently detected in the IgA^+^ PC compartment [26], and that PPs play a generative role in increasing clonal diversity rather than selecting antigen-specific clones in lower-order mammals [3].

Since the diversity of a complex microbiota poses a problem in defining specificity, investigators instead utilise immunisation, monocolonisation, and other gnotobiotic settings. In a series of oral immunisation experiments utilising CT coupled to a hapten, Bergqvist et al. could show a clearly detectable response to the hapten that was dominated by a few expanded B cell clones which were found in different inductive sites (mainly PPs) and distributed all along the LP in what they termed a ‘synchronised’ manner [18]. In the reversible colonisation experiments described above using auxotrophic bacteria, the evoked response was specific to the bacteria used for colonisation [15]. Using the same auxotrophic bacteria, Rollenske et al. found no significant increase in polyreactivity in microbiota-reactive IgA in GF mice primed with auxotrophic bacteria through the mucosa [27]. Both research groups showed preferential binding of membrane antigens in these settings [15, 27]. Dissecting the binding patterns of LP PC-derived IgA in monocolonised and gnotobiotic mice also revealed higher responses to strains present in the respective mice than to other closely related bacteria [28]. In a more functional approach, Donaldson et al. showed that the anchoring of *Bacteroides fragilis* in the mucus layer and the resistance to colonisation by other *Bacteroides* strains competing for the same niche depends on the induction of specific IgA towards capsular polysaccharides of the respective strain. Anchoring to the mucosa and colonisation resistance were abolished if the bacteria lacked the respective capsular polysaccharides, the mouse lacked IgA, or lacked the recombination-activating gene needed for B and T cell development [29].

All of this shows clearly detectable binding and even functional roles of IgA responses specific to the bacteria inducing them. This specificity is reflected in the role of microbiota in immune system maturation. When analysing mice colonised with a community of bacteria selected for their preferred presence in pre-weaning mice, Lubin et al. could show that those mice failed to accumulate peripheral Treg cells during weaning compared to mice with a complex microbiome and displayed higher levels of serum IgE [17]. Furthermore, mice colonised with this community were highly susceptible to infection by *Salmonella enterica* serovar Typhimurium resembling the higher susceptibility to enteric pathogens observed in infants [17]. This is specific for the early life community as another gnotobiotic community modelled after the adult mouse microbiome was able to confer protection against *Salmonella* infection [17, 30]. A higher susceptibility to infection with *Salmonella* was also observed in AID-KO mice [31]. The specific bacteria comprising a community and the responses of the host immune system are therefore likely involved in susceptibility to disease. For a more detailed analysis of IgA function, we refer the readers to Pabst and Nowosad [3] and Ng et al. [5].

### Results in complex microbial settings suggest resident microbiota driven B cell specificity

In specific pathogen-free (SPF) settings representing a complex homeostatic microbiota, Bunker et al. found that IgA^+^ PCs were enriched in microbiota reactivity and microbiota reactive monoclonal antibodies (mAbs) were enriched in polyreactivity over naïve B cells [26]. They showed that polyreactive and microbiota-reactive B cells are derived independently of GCs and bind a broad but well defined microbial fraction commonly targeting glycans [26] corroborated by the outer membrane targeting found by Rollenske et al. [27]. They also found polyreactive mAb derived from influenza infections as well as IgA derived from GF mice to bind a subset of microbiota similar to SPF IgA^+^ PCs [26]. In contrast, in a human dataset, Kabbert et al. found overall lower levels of polyreactivity in the mAbs tested and microbiota-binding capacity did not correlate with increased polyreactivity [32]. Furthermore, they showed that the majority of mAbs that were highly microbiota-reactive exhibited reduced binding capacities upon reversion of mutations to the inferred germline configuration of the antibodies [32]. Moreover, as mentioned before, it has been shown that in mice under SPF conditions, the IgA^+^ PC compartment is highly mutated and increases in diversity and mutational load over the lifetime of the mouse [33]. The specific, highly mutated repertoire was recalled when LP PC were selectively depleted through application of the proteasome inhibitor Bortezomib, hinting at an important role for PC recruited from a comparably stable set of specifically selected B cell clones [23, 33].

A functional role of SHM in the regulation of the microbiome was also found by Fagarasan et al. using AID knockout mice, which cannot mutate their BCRs [34]. They showed that AID-deficient mice had an altered small intestinal microbiome with anaerobic outgrowth, hyperplastic PPs, and ILFs. The hyperplastic PPs and ILFs decreased in size upon administration of antibiotics targeting outgrown anaerobic bacteria. The ILF hyperplasia was not seen in IgA-deficient mice where SHM is intact indicating a specific role for SHM rather than antibody isotype choice [34], which is also dependent on AID [8]. More broadly, Kawamoto et al. showed reduced bacterial diversity in mice with adaptive immune deficiencies (lacking T and B cells or lacking only T or B cells) [22]. They also showed that FoxP3^+^ T cells entering GCs select for a more diverse, spore-forming bacteria-rich microbiome. This microbiome, in turn, induces an immune response skewed towards IgA production and FoxP3^+^ T cell development when transferred into GF mice [22].

Approaching this question at the single GC level, Nowosad et al., using advanced imaging to visualize B cell selection in single GCs [10], found highly selected gaGCs in SPF mice [11]. Some of the antibodies produced from those GCs could bind to faecal bacteria of SPF mice and this binding was diminished upon reversion of the antibody to its inferred germline sequence, clearly evidencing specific selection and affinity maturation of commensal-specific B cells in gaGCs [11]. They even found an antibody binding with high affinity to a single bacterial species in a gnotobiotic mouse model. Importantly, none of the antibodies produced from single GCs show substantial polyreactivity [11].

There is clear evidence of antigen-specific selection in oral immunisation and in settings of reduced microbiota diversity, and we have initial evidence of this in full microbiome settings under steady-state conditions. There is still uncertainty regarding the contribution of germline-encoded polyreactive B cells and GC-derived antigen-specific B cells to the IgA^+^ B cell pool under steady-state conditions and their functional role in gut homeostasis.

### Clinical insight into the significance of the humoral immune response in the gut

The specific IgA and its interplay with the resident bacteria might play a role in the pathogenesis of inflammatory bowel diseases (IBD). IBD is believed to rely on an aberrant immune response and the resident complex microbiome is implicated in this response [35, 36]. Bacteria from patients with IBD that are highly coated in IgA can drive susceptibility to colitis in mouse models. Furthermore, in a mouse model of dysbiosis the disease driving bacteria are coated highly in IgA in a T cell dependent manner indicating a specific response [35]. One of those bacteria is segmented filamentous bacteria, that is known to induce T 17 helper cells [37] which are also implicated in IBD pathogenesis [36]. Antibodies expressed from IgA and IgG LP PCs of patients with IBD showed overall higher reactivity to faecal bacteria than healthy donors [32] and mucosal IgG^+^ cells are generally known to be expanded in IBD patients [36]. The pathological role of B cells in IBD is in line with a recent report investigating vedolizumab treatment in IBD where a reduction in gut-homing IgG^+^ and IgA^+^ PB and a reduced entry of naïve B and T cells into the GALT was demonstrated [38].

A similar discovery has been made in malnourished children. The transplantation of highly IgA coated bacteria into GF mice in combination with a nutrient deficient diet led to rapid weight loss when compared to mice transplanted with highly coated bacteria from controls [39]. Whilst the dependency on T cells was not assessed in this study, a correlation between a robust IgA response against pathogenic *Enterobacteriacae* early in life and better development outcomes was suggested. The authors furthermore could show a shift in the IgA coating of the bacteria most associated with malnourishment upon dietary intervention in affected children [39], demonstrating the responsiveness of the IgA system.

Finally, IgA function can be assessed in models of IgA-deficiency and selective IgA-deficiency (sIgAD) is the most common primary human immunodeficiency [5]. SIgAD is diagnosed by reduced serum IgA levels (< 0.07 g/L) and normal IgM and IgG levels after the age of 4 years [40]. Surprisingly most patients with sIgAD are asymptomatic. Nonetheless sIgAD has been linked to multiple diseases ranging from recurrent sinopulmonary infections and allergy to autoimmune diseases and IBD [5, 40]. Especially in celiac disease there is a stark overrepresentation of sIgAD patients [40]. The differences in clinical presentation might be due to the diverse pathogenesis of sIgAD with different aetiologies involved including potential combinations with other immunological disorders [40] and a partial compensation by IgM [5]. However, it has been shown that sIgAD patients have reduced bacterial species diversity, species richness and gene richness [41] and there might be a disruption in the spatial organisation of the resident commensal bacteria [5]. Additionally, the sIgAD microbiome is enriched for species associated with type 2 diabetes, obesity, and intestinal dysbiosis as well as for genes enconding antimicrobial resistance and proinflammatory potential [41].

The clinical implications of distinct IgA responses and IgA deficiency in multiple diseases implicate a nuanced IgA response to the resident microbiota [5]. The recent advances in the knowledge of selection in gaGCs coupled with in-depth analysis of IgA function and careful dissection of shifts in the microbiota will further improve our understanding of clinical implications of the gut associated humoral immune system in health and disease.

## Conclusion

**Fig. 1:**
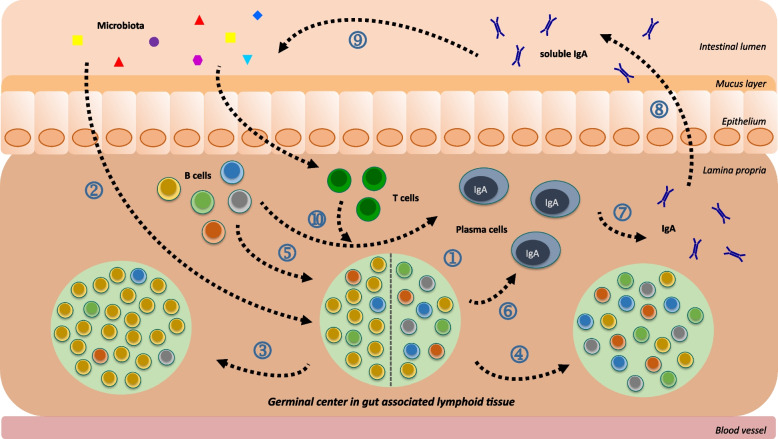
B cell selection at mucosal surfaces. The GC depicted with B cells in different colours to symbolise B cell clone diversity (1) following the idea of ‘brainbow’ [10] reacts to the antigen transported to it across the mucosal membrane (2). Depending on the circumstances the selection can be pronounced with one clone taking over the GC in a clonal burst (3) or the GC can remain diverse (4). The gaGCs also continuously become invaded by new B cells of unknown specificity (5). Ultimately the GC exports effector cells (6) of which PC produce IgA (7), which gets secreted into the lumen (8) and in turn affects the microbiota in a variety of ways (9) [3, 5]. In a T cell independent manner, the switch of naïve B cells to IgA^+^ PC may happen independent of the GC through induction by the presence of microbiota (10). The secreted IgA does not necessarily clear the inducing agent, as is usually the case in infectious settings. This leads to the ongoing production of IgA without termination of residency by microbiota (even potentially harmful species) [5]

The B cell response in the GALT is largely dominated by the IgA isotype [3]. Although IgA switching is preserved even under conditions of severe T cell deficiency, most LP IgA^+^ PC are dependent on the presence of functional T cells under physiological conditions [2, 3, 5, 23]. Additionally there might be a critical window of immune maturation during early life contributing to the B cell response in GALT [16]. In immunisation and monocolonisation models the occurrence of specific responses to inducing bacteria or antigens is more evident, exhibiting the potential of gaGCs to mount specific responses under these more defined conditions [11, 27, 29]. This becomes less clear within settings of complex microbial communities, though specific responses are indeed observed in human datasets [32] or in mice using single GC resolution [11]. Other studies have reported high levels of polyreactivity within the LP PC compartment independent of GCs [26]. Nonetheless, even under these complex conditions, important functional roles of B cell SHM have been described [5, 22, 29]. These complex interactions are reflected in the clinical presentation of several diseases. The observation of differential coating of bacteria with IgA in IBD and malnourishment, the differences in mutational patterns in TG2-reactive B cells, heightened susceptibility to gastrointestinal infection and a variety of outcomes for patients with sIgAD showcase the nuanced B cell response in the GALT and its clinical implications. As summarised in Fig. 1 the B cell response at mucosal surfaces consists of a delicate interplay of many dynamic factors, including the microbiota and continuous B cell influx, that in turn might be further influenced by the IgA output of the GCs. Therefore, when dissecting the rules underlying these responses in health and disease, it is important to consider not only each component, but also its effect on the other components, especially in reductionist systems.

## Data Availability

No datasets were generated or analysed during the current study.

## Abbreviations

AID: Activation-induced cytidine deaminase
BCR: B cell receptor
CT: Cholera toxin
Foxp3 T cell: Forkhead box P3 T cell
gaGC: Gut associated germinal centre
GALT: Gut associated lymphoid tissue
GC: Germinal centre
GF: Germ-free
IBD: Inflammatory bowel diseases
Ig: Immunoglobulin
ILF: Isolated lymphoid follicle
LP: Lamina propria
mAb: Monoclonal Antibody
mLN: Mesenteric lymph nodes
PB: Plasmablasts
PC: Plasma cells
PP: Peyer’s patches
SHM: Somatic hypermutation
sIgAD: Selective IgA-deficiency
SPF: Specific pathogen free
TG2: Transglutaminase 2
Treg cell: Regulatory T cell
V-, D-, J-segment: Variable- (V-), diversity- (D-) and joining- (J-) segment

## References

[1] Sender R, Weiss Y, Navon Y et al (2023) The total mass, number, and distribution of immune cells in the human body. Proc Natl Acad Sci 120:e2308511120. 10.1073/pnas.2308511120 37871201 10.1073/pnas.2308511120 PMC10623016

[2] Fagarasan S, Kawamoto S, Kanagawa O, Suzuki K (2010) Adaptive Immune Regulation in the Gut: T Cell-Dependent and T Cell-Independent IgA Synthesis. Annu Rev Immunol 28:243–273. 10.1146/annurev-immunol-030409-101314 20192805 10.1146/annurev-immunol-030409-101314

[3] Pabst O, Nowosad CR (2023) B cells and the intestinal microbiome in time, space and place. Semin Immunol 69:101806. 10.1016/j.smim.2023.101806 37473559 10.1016/j.smim.2023.101806

[4] Kubinak JL, Round JL (2016) Do antibodies select a healthy microbiota? Nat Rev Immunol 16:767–774. 10.1038/nri.2016.114 27818504 10.1038/nri.2016.114 PMC9004535

[5] Ng KW, Hobbs A, Wichmann C et al (2022) B cell responses to the gut microbiota. Adv Immunol 155:95–131. 10.1016/bs.ai.2022.08.003 36357013 10.1016/bs.ai.2022.08.003

[6] Sutherland DB, Suzuki K, Fagarasan S (2016) Fostering of advanced mutualism with gut microbiota by Immunoglobulin A. Immunol Rev 270:20–31. 10.1111/imr.12384 26864102 10.1111/imr.12384

[7] Mesin L, Ersching J, Victora GD (2016) Germinal center B cell dynamics. Immunity 45:471–482. 10.1016/j.immuni.2016.09.001 27653600 10.1016/j.immuni.2016.09.001 PMC5123673

[8] Victora GD, Nussenzweig MC (2022) Germinal Centers. Annu Rev Immunol 40:413–442. 10.1146/annurev-immunol-120419-022408 35113731 10.1146/annurev-immunol-120419-022408

[9] Victora GD, Schwickert TA, Fooksman DR et al (2010) Germinal Center dynamics revealed by multiphoton microscopy with a photoactivatable fluorescent reporter. Cell 143:592–605. 10.1016/j.cell.2010.10.032 21074050 10.1016/j.cell.2010.10.032 PMC3035939

[10] Tas JMJ, Mesin L, Pasqual G et al (2016) Visualizing antibody affinity maturation in germinal centers. Science 351:1048–1054. 10.1126/science.aad3439 26912368 10.1126/science.aad3439 PMC4938154

[11] Nowosad CR, Mesin L, Castro TBR et al (2020) Tunable dynamics of B cell selection in gut germinal centres. Nature 588:321–326. 10.1038/s41586-020-2865-9 33116306 10.1038/s41586-020-2865-9 PMC7726069

[12] Casola S, Otipoby KL, Alimzhanov M et al (2004) B cell receptor signal strength determines B cell fate. Nat Immunol 5:317–327. 10.1038/ni1036 14758357 10.1038/ni1036

[13] De Carvalho RVH, Ersching J, Barbulescu A et al (2023) Clonal replacement sustains long-lived germinal centers primed by respiratory viruses. Cell 186:131-146.e13. 10.1016/j.cell.2022.11.031 36565697 10.1016/j.cell.2022.11.031 PMC9870066

[14] Hägglöf T, Cipolla M, Loewe M et al (2023) Continuous germinal center invasion contributes to the diversity of the immune response. Cell 186:147-161.e15. 10.1016/j.cell.2022.11.032 36565698 10.1016/j.cell.2022.11.032 PMC9825658

[15] Hapfelmeier S, Lawson MAE, Slack E et al (2010) Reversible microbial colonization of germ-free mice reveals the dynamics of IgA immune responses. Science 328:1705–1709. 10.1126/science.1188454 20576892 10.1126/science.1188454 PMC3923373

[16] Vergani S, Muleta KG, Da Silva C et al (2022) A self-sustaining layer of early-life-origin B cells drives steady-state IgA responses in the adult gut. Immunity 55:1829-1842.e6. 10.1016/j.immuni.2022.08.018 36115337 10.1016/j.immuni.2022.08.018

[17] Lubin J-B, Green J, Maddux S et al (2023) Arresting microbiome development limits immune system maturation and resistance to infection in mice. Cell Host Microbe 31:554-570.e7. 10.1016/j.chom.2023.03.006 36996818 10.1016/j.chom.2023.03.006 PMC10935632

[18] Bergqvist P, Stensson A, Hazanov L et al (2013) Re-utilization of germinal centers in multiple Peyer’s patches results in highly synchronized, oligoclonal, and affinity-matured gut IgA responses. Mucosal Immunol 6:122–135. 10.1038/mi.2012.56 22785230 10.1038/mi.2012.56

[19] Bunker JJ, Flynn TM, Koval JC et al (2015) Innate and adaptive humoral responses coat distinct commensal bacteria with immunoglobulin A. Immunity 43:541–553. 10.1016/j.immuni.2015.08.007 26320660 10.1016/j.immuni.2015.08.007 PMC4575282

[20] Macpherson AJ, Gatto D, Sainsbury E et al (2000) A primitive T cell-independent mechanism of intestinal mucosal IgA responses to commensal bacteria. Science 288:2222–2226. 10.1126/science.288.5474.2222 10864873 10.1126/science.288.5474.2222

[21] Biram A, Strömberg A, Winter E et al (2019) BCR affinity differentially regulates colonization of the subepithelial dome and infiltration into germinal centers within Peyer’s patches. Nat Immunol 20:482–492. 10.1038/s41590-019-0325-1 30833793 10.1038/s41590-019-0325-1

[22] Kawamoto S, Maruya M, Kato LM et al (2014) Foxp3+ T Cells Regulate Immunoglobulin A Selection and Facilitate Diversification of Bacterial Species Responsible for Immune Homeostasis. Immunity 41:152–165. 10.1016/j.immuni.2014.05.016 25017466 10.1016/j.immuni.2014.05.016

[23] Lindner C, Thomsen I, Wahl B et al (2015) Diversification of memory B cells drives the continuous adaptation of secretory antibodies to gut microbiota. Nat Immunol 16:880–888. 10.1038/ni.3213 26147688 10.1038/ni.3213

[24] Stamnaes J, Sollid LM (2015) Celiac disease: autoimmunity in response to food antigen. Semin Immunol 27:343–352. 10.1016/j.smim.2015.11.001 26603490 10.1016/j.smim.2015.11.001

[25] Bemark M, Sale JE, Kim H-J et al (2000) Somatic hypermutation in the Absence of DNA-dependent protein kinase catalytic subunit (DNA-Pkcs) or recombination-activating gene (Rag)1 Activity. J Exp Med 192:1509–1514. 10.1084/jem.192.10.1509 11085752 10.1084/jem.192.10.1509 PMC2193187

[26] Bunker JJ, Erickson SA, Flynn TM, et al (2017) Natural polyreactive IgA antibodies coat the intestinal microbiota. Science 358:eaan6619. 10.1126/science.aan6619 10.1126/science.aan6619 PMC5790183 28971969

[27] Rollenske T, Burkhalter S, Muerner L et al (2021) Parallelism of intestinal secretory IgA shapes functional microbial fitness. Nature 598:657–661. 10.1038/s41586-021-03973-7 34646015 10.1038/s41586-021-03973-7

[28] Yang C, Chen-Liaw A, Spindler MP, et al (2022) Immunoglobulin A antibody composition is sculpted to bind the self gut microbiome. Sci Immunol 7:eabg3208. 10.1126/sciimmunol.abg3208 10.1126/sciimmunol.abg3208 PMC9421563 35857580

[29] Donaldson GP, Ladinsky MS, Yu KB et al (2018) Gut microbiota utilize immunoglobulin A for mucosal colonization. Science 360:795–800. 10.1126/science.aaq0926 29724905 10.1126/science.aaq0926 PMC5973787

[30] Brugiroux S, Beutler M, Pfann C et al (2016) Genome-guided design of a defined mouse microbiota that confers colonization resistance against Salmonella enterica serovar Typhimurium. Nat Microbiol 2:16215. 10.1038/nmicrobiol.2016.215 27869789 10.1038/nmicrobiol.2016.215

[31] Hara S, Sasaki T, Satoh-Takayama N et al (2019) Dietary Antigens Induce Germinal Center Responses in Peyer’s Patches and Antigen-Specific IgA Production. Front Immunol 10:2432. 10.3389/fimmu.2019.02432 31681315 10.3389/fimmu.2019.02432 PMC6803481

[32] Kabbert J, Benckert J, Rollenske T et al (2020) High microbiota reactivity of adult human intestinal IgA requires somatic mutations. J Exp Med 217:e20200275. 10.1084/jem.20200275 32640466 10.1084/jem.20200275 PMC7526496

[33] Lindner C, Wahl B, Föhse L et al (2012) Age, microbiota, and T cells shape diverse individual IgA repertoires in the intestine. J Exp Med 209:365–377. 10.1084/jem.20111980 22249449 10.1084/jem.20111980 PMC3280880

[34] Fagarasan S, Muramatsu M, Suzuki K et al (2002) Critical roles of activation-induced cytidine deaminase in the Homeostasis of Gut Flora. Science 298:1424–1427. 10.1126/science.1077336 12434060 10.1126/science.1077336

[35] Palm NW, de Zoete MR, Cullen TW et al (2014) Immunoglobulin a coating identifies colitogenic bacteria in inflammatory bowel disease. Cell 158:1000–1010. 10.1016/j.cell.2014.08.006 25171403 10.1016/j.cell.2014.08.006 PMC4174347

[36] Castro-Dopico T, Colombel J-F, Mehandru S (2020) Targeting B cells for inflammatory bowel disease treatment: back to the future. Curr Opin Pharmacol 55:90–98. 10.1016/j.coph.2020.10.002 33166872 10.1016/j.coph.2020.10.002 PMC7894973

[37] Ivanov II, Atarashi K, Manel N et al (2009) Induction of intestinal Th17 cells by segmented filamentous bacteria. Cell 139:485–498. 10.1016/j.cell.2009.09.033 19836068 10.1016/j.cell.2009.09.033 PMC2796826

[38] Canales-Herrerias P, Uzzan M, Seki A, et al (2024) Gut-associated lymphoid tissue attrition associates with response to anti-α4β7 therapy in ulcerative colitis. Sci Immunol 9:eadg7549. 10.1126/sciimmunol.adg7549 10.1126/sciimmunol.adg7549 PMC11140591 38640252

[39] Kau AL, Planer JD, Liu J, et al (2015) Functional characterization of IgA-targeted bacterial taxa from undernourished Malawian children that produce diet-dependent enteropathy. Sci Transl Med 7:276ra24. 10.1126/scitranslmed.aaa4877 10.1126/scitranslmed.aaa4877 PMC4423598 25717097

[40] Yazdani R, Azizi G, Abolhassani H, Aghamohammadi A (2017) Selective IgA Deficiency: epidemiology, pathogenesis, clinical phenotype, diagnosis, prognosis and management. Scand J Immunol 85:3–12. 10.1111/sji.12499 27763681 10.1111/sji.12499

[41] Moll JM, Myers PN, Zhang C et al (2021) Gut Microbiota perturbation in IgA deficiency is influenced by IgA-Autoantibody status. Gastroenterology 160:2423-2434.e5. 10.1053/j.gastro.2021.02.053 33662387 10.1053/j.gastro.2021.02.053

